# Culture Effects on the Chinese Version Boston Naming Test Performance and the Normative Data in the Native Chinese-Speaking Elders in Mainland China

**DOI:** 10.3389/fneur.2022.866261

**Published:** 2022-05-13

**Authors:** Yan Li, Yuchen Qiao, Fen Wang, Cuibai Wei, Rui Wang, Hongmei Jin, Bingxin Xie, Jing You, Jianping Jia, Aihong Zhou

**Affiliations:** Innovation Center for Neurological Disorders and Department of Neurology, National Clinical Research Center for Geriatric Diseases, Xuanwu Hospital, Capital Medical University, Beijing, China

**Keywords:** Boston Naming Test, confrontation naming, cross-cultural, lexical familiarity, normative data, Chinese population, elders

## Abstract

**Background:**

The Chinese version of Boston Naming Test (BNT-C) is administered in China widely. However, the neuropsychological parameter of BNT-C in native Chinese-speaking elders in mainland China has not been explored systematically. The aim of this study was to explore cultural influences on BNT-C performance and establish norms among native Chinese-speaking elders in Beijing.

**Methods:**

A total of 161 native, Chinese-speaking, cognitively normal elders aged ≥55 years were enrolled from various communities in Beijing. The BNT-C was conducted on all the participants. The internal consistency, participants' familiarity, and naming accuracy were analyzed and compared with data from Chinese areas outside the mainland and from American published previously. The influencing factors and stratified norms for BNT-C were established.

**Results:**

The BNT-C showed good internal consistency (α = 0.738). Strong correlation between naming accuracy and object familiarity was found (*r* = 0.962, *P* < 0.001). Participants' familiarity and correct naming rate for many items were notably different between the Chinese-speaking elders and English-speaking elders in America. The difference in some items' correct naming rate also existed between Beijing, Taiwan, and Hongkong. Higher education was associated with higher scores, whereas age and gender had no effect on BNT-C performance. The recommended norms of total naming scores for elders with education ≤ 9 and >9 years were 16 and 23, respectively.

**Conclusion:**

The participants' familiarity with BNT items differed between different cultures, which further affected the naming accuracy and total scores. The education stratified norms established here are helpful for the better application of BNT-C in mainland China.

## Introduction

The Boston Naming Test (BNT) was compiled by Goodglass and Kaplan in 1983, which is composed of 60-line drawings of objects and animals, ranging from very familiar objects (trees and pencils) to unfamiliar objects (Sphinx and scaffolding) (1). By far, it is the most widely used confrontational naming test in the world and provides valuable diagnostic information for patients with aphasia and other cognitive-linguistic impairments from stroke (2), head injury (3), and neurodegenerative diseases such as frontotemporal dementia (4).

The BNT was originally designed for English-speaking people in North America. Individuals' familiarity with BNT items differs among culture, populations, and countries, which likely affect the naming performance and total scores (5). Therefore, it is important to explore the item familiarities and establish BNT norms according to local cultural and linguistic populations when used outside North America (5). So far, BNT has been adapted to many languages, including Danish (6), Spanish (7), Chinese (8), Dutch (9), Korean (10), French Canadian (11), Greek (12), Italian (13), Malay (14), and Swedish (15). Among the various versions of the BNT in different languages, some of them adopted the original English items (6, 15, 16) and others made adjustments or replacements to some items to adapt to the local cultural background (12, 17, 18). Even in other English-speaking countries outside America, such as Australia (19), BNT also had to be adapted to local populations.

The Chinese version of BNT (BNT-C) was developed by Hongkong scholars by selecting 30 items from the original English version without item adjustment and replacement (20). The authors proved that BNT-C successfully distinguished naming impairment in Cantonese-speaking patients with a head injury from a normal control group. Since then, BNT-C has been widely used across China. Due to cultural differences, people's familiarity with BNT items varies between the Chinese and Caucasian populations. For example, the igloo and harp are relatively unfamiliar to the Chinese, while the abacus, which is considered the most difficult for Americans, is well-known to many Chinese. Consequently, the influential factors and norm data of the BNT-C may differ markedly from those in the west. However, there has been no study exploring the familiarity of the BNT-C. The per-item correct naming rate and striated norm of BNT-C in China mainland have not been evaluated and reported either. In this study, we administered BNT-C to 161 Chinese-speaking community elders in Beijing. The participants' familiarity and naming performance for each BNT-C item were determined and compared with those from American (21) as well as other Chinese areas (Taiwan and Hongkong) published previously (5, 20). The correlations between naming accuracy and familiarity were further explored. The effects of demographic variables (gender, age, and education) on naming performance were examined, and stratified norms were established considering significant influential factors.

## Methods

This study was conducted from January 2018 to November 2019 at five communities in Beijing, China. The study protocol was approved by the Ethics Review Board of the Xuanwu Hospital, Beijing Capital Medical University. Written informed consent was obtained from all the participants.

### Subjects

The participants were recruited from community volunteers in Beijing. The eligible samples for inclusion were (1) 55–85 years old, (2) native Chinese speakers, and (3) the Mini-Mental State Examination (MMSE) ≥24 (22). Neurologists interviewed all the subjects. Any individuals with a history of psychiatric or central nervous system diseases, hearing loss, learning disability, and any other condition that was likely to have an influence on performance in the BNT were excluded.

### Chinese Version of BNT

The BNT-C used in this study consists of 30 items (**Table 2**) selected from the original 60 items without item adaption (20). The order of presentation followed the original sequence, and the design of the stimulus cards was identical to that of the original pictures (20).

### Procedures and Scoring

The BNT-C was administered to all the participants by trained raters as described by Cheung RW who, together with his colleagues, developed the BNT-C test (20). All 30 cards bearing the line-drawing objects or animals were presented to the elders in a fixed order. Participants were instructed to name each object depicted on the cards. If the participant named one item correctly, one point was awarded and it was recorded among “scores of spontaneous naming (SN).” The examiner then proceeded to the next item. If the participant gave a wrong response or gave no response within 20 s, the participant's response was recorded in detail and a standard semantic cue was provided (e.g., “it is a plant” for “tree”). A semantic cue was designed for each item as in the original version of BNT. If the participant gave the correct answer, one point was awarded and it was recorded among “scores after semantic cue (SC)” (20). In the original BNT, if the participants failed the semantic cue, then a phonemic cueing was supplied. Given that Chinese is a logographic language and the names of most objects consist of one sound, BNT-C adopted a multiple-choice recognition task including the target response, name of an object similar to the target in function (e.g., “cow” for “camel”), and name of an object similar to the target in appearance (e.g., “mountain” for “camel”). The participant's response was recorded but no credit was given for choosing naming. The total score of BNT-C ranged from 0 to 30 (“SN” plus “SC”), the higher indicating the better naming ability.

### Familiarity Rating

The participants were instructed to rate each item of BNT-C for familiarity based on how usual or unusual the objects were in their experience, using a 5-point scale ranging from 1 (very unfamiliar) to 5 (very familiar). Familiarity was defined as “the degree to which you come in contact with or think about the object.”

### Statistical Analysis

Statistical analyses were performed with SPSS version 24.0 (SPSS Inc., Chicago, Ill., USA). Demographic and neuropsychological data were presented as mean ± SD or number and percentage. The mean data between two groups were analyzed using an independent-sample *t*-test or χ^2^ test (chi-square). The internal consistency of BNT-C was assessed by Cronbach's alpha. Pearson's correlation coefficient was used to determine the correlation between naming accuracy and familiarity. A multiple linear regression analysis was used to explore the influence of age, gender, and education on BNT-C performance. For all tests, *P* < 0.05 was considered statistically significant.

## Result

In total, 161 cognitively normal elders, 74 men (46.0%) and 87 women (54.0%), were recruited for the current study. The average age was 71 years (range: 55–85 years). The average period of formal education was 11.4 years (range: 1–19 years). The average MMSE score was 28.03 (range: 25–31). Demographic data and MMSE scores stratified by education were presented in Table 1.

**Table 1:** Demographic and neuropsychological data.

	**Total**	**Education (years)**		**t**	***P*** **-value**
		**≤9**	**>9**		
Number	161	72	89	/	
Male: female ratio	74:87	31:41	43:46	0.443	0.506
Age, years	71.00 ± 6.29	70.70 ± 6.69	71.19 ± 6.06	−0.414	0.680
Education, years	11.41 ± 3.54	7.74 ± 1.46	13.76 ± 2.22	15.245	<0.001
MMSE	28.03 ± 1.65	27.45 ± 1.90	28.43 ± 1.33	−3.058	0.003
BNT-C scores (SN)	23.83 ± 3.37	21.79 ± 3.75	25.14 ± 2.33	−5.488	<0.001
BNT-C scores (TS)	25.26 ± 3.10	23.36 ± 3.65	26.47 ± 1.88	−5.405	<0.001
BNT-C cut-off score (SN)		14	20		
BNT-C cut-off score (TS)		16	23		

*MMSE, Mini-Mental State Examination; BNT-C, the Chinese version of the Boston Naming Test; SN, scores of spontaneous naming; TS, total scores (“scores of spontaneous naming” plus “scores after semantic cue”)*.

### Internal Consistency of the BNT-C

The 30 items composing the BNT-C showed a high-internal reliability coefficient (α = 0.738). Every item in the BNT-C was positively correlated with the total score and contributed positively to Cronbach's α for the total score.

### Per Item Familiarity and Correct Naming Rate of BNT-C

Participants' familiarities for each BNT-C item were recorded and compared with those from a study that rated the 60 BNT pictures on familiarity in 30 elder native English speakers in America (21). However, the study adopted a scale ranging from one (not at all familiar) to seven (very familiar) instead of one to five. To facilitate comparison, we multiplied their results by five-sevenths. The average familiarity of Chinese in our study was 4.33, but it was 4.94 in the American residents. Our elders rated not familiar (<4) in seven items (igloo, harp, pyramid, seahorse, dart, cactus, and trellis) and rated familiar or very familiar in the remaining 23 items. American elders rated all 30 items as familiar or very familiar. Further analysis into items with a familiarity difference >0.5 showed that Americans were much more familiar with igloos than Chinese (4.98 vs. 1.95). They were also more familiar with harp (5 vs. 3.07), pyramid (4.98 vs. 3.35), cactus (5 vs. 3.79), dart (4.86 vs. 3.71), seahorse (4.81 vs. 3.67), and trellis (4.93 vs. 3.83). The familiarities with rhinoceros, harmonica, tongs, protractor, and tripod were also 0.5 points higher in the Americans than in the Chinese residents. Abacus was the only item that the Chinese residents were more familiar with than the Americans (4.80 vs. 4.55) (Table 2).

**Table 2:** Per-item familiarity and correct naming rate of BNT-C in our study and other studies.

**Item**	**Original item No.**	**Item**	**Familiarity**		**Correct (%)**			
**No**.			**BJ**	**USA (21)**	**BJ**	**HK (20)**	**TW (5)**	**USA (23)**
Number			161	30	161	77	264	60
Age range			55–85	56–86	55–85	23–79	60–92	40–78
Education, years			11.4	15.0	11.4	9.7	12.5	13.9
SN			N/A	N/A	23.8 (3.4)	24.9 (3.0)	24.7 (3.9)	N/A
TS			N/A	N/A	25.2 (3.1)	26.7 (2.8)	N/A	54.5
1	2	Tree	4.84	5.00	100.0	100.0	100.0	100.0
2	3	Pencil	4.90	5.00	100.0	100.0	100.0	100.0
3	6	Scissors	4.92	5.00	100.0	100.0	100.0	98.3
4	8	Flowers	4.79	5.00	100.0	100.0	100.0	98.3
5	9	Saw	4.66	5.00	98.1	100.0	98.1	100.0
6	12	Broom	4.90	5.00	89.3	94.8	98.1	100.0
7	14	Mushroom	4.84	5.00	100.0	67.5	97.7	93.3
8	15	Hanger	4.91	5.00	99.0	100.0	99.2	100.0
9	16	Wheelchair	4.75	5.00	91.4	92.2	96.2	100.0
10	17	Camel	4.62	4.88	99.0	98.7	99.2	98.3
11	21	Racquet	4.76	5.00	100.0	97.4	98.1	100.0
12	22	Snail	4.60	4.95	98.1	90.9	96.2	100.0
13	24	Seahorse	3.67	4.81	63.1	90.9	82.6	91.7
14	25	Dart	3.71	4.86	61.4	83.1	73.9	98.3
15	30	Harmonica	4.24	5.00	80.6	83.1	84.5	86.7
16	31	Rhinoceros	4.13	4.95	88.4	81.8	93.6	83.0
17	33	Igloo	1.95	4.98	3.9	20.8	60.6	98.3
18	36	Cactus	3.79	5.00	71.8	85.7	86.4	100.0
19	37	Escalator	4.72	5.00	94.2	97.4	97.3	100.0
20	38	Harp	3.07	5.00	43.1	54.5	68.9	100.0
21	42	Stethoscope	4.67	5.00	95.2	83.1	87.9	96.7
22	43	Pyramid	3.35	4.98	70.9	83.1	79.5	95.0
23	46	Funnel	4.68	5.00	96.1	98.7	92.0	95.0
24	47	Accordion	4.57	4.95	96.1	77.9	84.8	91.7
25	50	Compass	4.63	4.91	93.2	90.9	92.0	46.7
26	52	Tripod	4.23	4.84	74.8	63.6	80.3	81.7
27	54	Tongs	4.20	4.95	83.5	81.8	96.6	81.7
28	57	Trellis	3.83	4.93	57.3	41.6	84.5	88.3
29	59	Protractor	4.16	4.81	73.8	33.8	44.7	35.0
30	60	Abacus	4.80	4.55	97.1	98.7	99.6	55.0

*BNT-C, the Chinese version of the Boston Naming Test; “Original Item No.” refers to the original BNT (1). Values for education and BNT are means. N/A, Not available; SN, scores of spontaneous naming; TS, total scores (“scores of spontaneous naming” plus “scores after semantic cue”); BJ, Beijing; HK, Hongkong; TW, Taiwan*.

Per item correct naming rates for the 30 BNT-C items were recorded and compared with data from American normal elders (23) and other Chinese normal elders outside the mainland [Taiwan (5) and Hong Kong (20)]. The correct naming rate difference between populations >20% was considered significant (5) (Table 2). Compared with the American residents, Beijing elders performed better in naming for compass, abacus, and protractor but performed worse in naming igloo, harp, dart, trellis, seahorse, cactus, and pyramid. The naming accuracy gap of igloo, harp, dart, protractor, compass, and abacus between the Chinese and the Americans was close to or >40%. Only 3.9% of the Beijing elders named igloo correctly compared with 98% of the American residents. In contrast, American residents got 55% and 46.7% correct naming rates for abacus and compass, respectively, lower than those of the Beijing residents (97.1 and 93.2%, respectively). Compared with the Hong Kong elders, the Beijing residents performed better in naming mushroom and protractor but worse in seahorse and dart. The Beijing residents named better in protractor and worse in seahorse, igloo, harp, and trellis than the Taiwan elders. Compared with the American elders, the Beijing, Taiwan, and Hong Kong elders were consistently good in naming compass and abacus but consistently worse in igloo, dart, and harp. All populations performed well in naming tree, pencil, scissors, flowers, racquet, hanger, camel, saw, snail, funnel, escalator, wheelchair, with a correct rate of >90%.

Pearson correlation analysis was used to explore the correlation between correct naming and familiarity. The Pearson correlation coefficient was 0.962 (*P* < 0.001), which indicated that naming accuracy was highly correlated with familiarity.

### Influential Factors and Stratified Norms for BNT-C

Multiple linear regression analysis was used to explore the effect of age, gender, and education on BNT-C performance (Table 3). No significant correlations between age or gender and BNT-C scores were found. The analysis identified that education strongly correlated with BNT-C performance. Therefore, the subjects were subgrouped into the following four educational categories: (1) ≤ 6 years (*n* = 32); (2) 7–9 years (*n* = 40); (3) 10–12 years (*n* = 42), and (4) ≥13 years (*n* = 47). According to the *post hoc* analysis, there were no significant differences between ≤ 6 years and 7–9 years of education and between 10–12 years and ≥13 years of education in scores for spontaneous naming (≤6 vs. 7–9 years: 20.29 ± 3.95 vs. 22.66 ± 3.31; 10–12 vs. ≥13 years: 25.00 ± 2.28 vs. 25.30 ± 2.37) and total scores after semantic cueing (≤6 vs. 7–9 years: 22.29 ± 3.58 vs. 23.94 ± 3.49; 10–12 vs. ≥13 years: 26.38 ± 1.83 vs. 26.65 ± 1.85). Accordingly, we subgrouped the participants into two education level groups (≤9 and >9 years) eventually.

**Table 3:** Results of multiple linear regression analysis of gender, age, and education on BNT-C score.

	**Unstandardize B**	**Coefficients std. error**	**Standardized coefficients** **beta**	**t**	***P*** **-value**
(Constant)	24.466	3.949		6.195	<0.001
Gender	−1.043	0.587	−0.180	−1.778	0.079
Age	−0.002	0.055	−0.004	−0.041	0.967
Education	1.854	0.632	0.310	2.936	0.004

*BNT-C, the Chinese version of the Boston Naming Test*.

The naming accuracy was significantly higher in the high education group (>9 years) than in the lower education group (≤9 years) in seahorse (77.00 vs. 42.90%, *P* < 0.001), dart (68.90 vs. 47.60%, *P* = 0.031), rhinoceros (95.10 vs. 78.60%, *P* = 0.010), harp (54.10 vs. 26.20%, *P* = 0.005), pyramid (86.90 vs. 47.60%, *P* < 0.001), compass (100.00 vs. 83.30%, *P* = 0.004), tripod (90.20 vs. 52.40%, *P* < 0.001), tongs (91.80 vs. 71.40%, *P* = 0.006), and protractor (85.20 vs. 57.10%, *P* = 0.001) (Table 4). Consequently, the high education group achieved better spontaneous naming scores (25.14 ± 2.33 vs. 21.79 ± 3.75, *P* < 0.001) and total scores (26.47 ± 1.88 vs. 23.36 ± 3.65, *P* < 0.001) than the lower education group (Table 1). We chose “mean-2^*^SD” as the recommended cut-off value. For the individuals with formal education >9 years, the appropriate cutoff was 20 for spontaneous naming scores and 23 for total scores. For the individuals with formal education ≤ 9 years, the cutoff scores were 14 and 16, respectively.

**Table 4:** Percentage of correct BNT-C between two different education levels.

**Item**	**Correct (%)**		**χ^2^**	***P*** **-value**
	**≤9 years**	**>9 years**		
Tree	100.00	100.00	–	–
Pencil	100.00	100.00	–	–
Scissors	100.00	100.00	–	–
Flowers	100.00	100.00	–	–
Saw	95.20	100.00	2.96	0.164
Broom	88.10	90.20	0.00	0.992
Mushroom	100.00	100.00	–	–
Hanger	97.60	100.00	1.47	0.408
Wheelchair	95.20	88.50	0.69	0.406
Camel	100.00	98.40	0.70	1.000
Racquet	100.00	100.00	–	–
Snail	97.60	98.40	0.07	1.000
Seahorse	42.90	77.00	12.49	<0.001
Dart	47.60	68.90	4.68	0.031
Harmonica	76.20	83.60	0.87	0.35
Rhinoceros	78.60	95.10	6.59	0.010
Igloo	4.80	3.30	0.00	1.000
Cactus	64.30	77.00	2.00	0.157
Escalator	90.50	96.70	0.81	0.367
Harp	26.20	54.10	7.92	0.005
Stethoscope	92.90	96.70	0.19	0.667
Pyramid	47.60	86.90	18.58	<0.001
Funnel	92.90	98.40	0.81	0.367
Accordion	92.90	98.40	0.81	0.367
Compass	83.30	100.00	8.44	0.004
Tripod	52.40	90.20	18.82	<0.001
Tongs	71.40	91.80	7.49	0.006
Trellis	54.80	59.00	0.18	0.668
Protractor	57.10	85.20	10.16	0.001
Abacus	100.00	95.10	0.74	0.388

*BNT-C, the Chinese version of the Boston Naming Test*.

## Discussion

BNT was originally developed for the English-speaking populations in America. Because of its practicability and simplicity, many scholars have translated and adapted BNT for people with different languages and cultural backgrounds. BNT-C was composed by selecting 30 items from the original English version and is widely used in China. However, the psychometrics of the BNT-C has been rarely reported on and evaluated in the native Chinese-speaking elderly in the mainland. This study explored the internal consistency, per item familiarity, and correct naming rate of the BNT-C for the first time and generated striated norms for the Chinese-speaking elders in mainland China. We found good internal consistency in BNT-C, indicating that the items of this test reliably measure the same construct. Compared with the Americans, the Chinese elders were significantly less familiar with many items in the BNT-C and achieved low naming accuracy and total scores. We identified a positive correlation between education and BNT-C performance and established norms according to different education levels.

### Object Familiarity and Naming Accuracy of BNT-C in Different Cultural Backgrounds

BNT consists of 60 items for participants to name, including common things such as beds and trees, as well as uncommon objects such as pyramids and sphynx. People vary in their familiarities with each item due to different cultural backgrounds, life experiences, and education levels. However, familiarity data have been rarely evaluated and reported previously both in China and other countries. The current study found that the Chinese elders were not familiar with igloo, harp, pyramid, seahorse, dart, cactus, and trellis, which are not common in daily life or ordinary readings in China. Ferraro et al. rated the BNT pictures on familiarity in 30 elder native English speakers in America (21). As expected, the Chinese residents were less familiar with many items than the American residents. Most Chinese elders were very unfamiliar with igloo, which was very familiar to the Americans. In contrast, the Chinese residents were more familiar with abacus which was less familiar to the Americans. Many Chinese elders used the abacus for calculation in their early lives. The discrepancies in familiarities for BNT items truly reflected the cultural difference between China and America. Besides BNT-C, there were many adapted versions outside North America. Most modified versions replaced part test items according to local cultures (12, 17, 18). It seems appropriate to consider the possibility of replacing items that are not suitable for the Chinese individuals with more culturally representative items in the adapted Chinese BNT version. The development of new test items was outside the scope of this study, but future research should certainly address this consideration.

A previous study indicated that picture familiarity facilitates the processing of BNT word representations (24). It is expected that people likely name objects correctly when they are familiar with them. Consistently, we found a strong positive correlation between naming accuracy and item familiarity in the BNT-C performance. This correlation further explained the difference in per item correct naming rate between the Chinese and American residents. The American residents performed better in naming igloo, cactus, harp, and pyramid which they were more familiar with than the Chinese. In contrast, they perfomed significantly worse in naming abacus which they were least familiar with. The results further demonstrated how familiarity influenced naming accuracy.

BNT-C was developed by the Hong Kong scholars and was used in Taiwan and the mainland widely. Compared with people from Hong Kong or Taiwan, Beijing elders performed better in naming protractor but worse in seahorse, dart, igloo, and harp. This was probably because the residents in Hong Kong and Taiwan were more influenced by western culture than the Chinese mainland residents. Compared with the Americans, the Chinese people including Beijing, Taiwan, and Hong Kong consistently performed better in naming compass and abacus but consistently worse in igloo, dart, and harp. This consistency reflected the long-term differences between the Chinese culture and the American culture.

Hobson et al. (25) showed a reliable creation of an estimated 60-item BNT score from administrations of the 30-item BNT by multiplying the obtained score by two. By this method, the total BNT scores from our study and those from Hong Kong were multiplied by the two to allow comparisons across different studies. The calculated total scores (“scores of spontaneous naming” plus “scores after semantic cuing”) for elders in Beijing (estimated BNT score of 50) were lower than that in Hong Kong (20) (estimated BNT score of 53) and America (23) (estimated BNT score of 55). Combining the familiarity differences and the correlation between familiarity and naming accuracy, we could infer that cultural background influences participants' familiarities with BNT items, which affects the naming accuracy further and impact the total scores ultimately. However, it should be noted that the per-item familiarity and correct naming rate of BNT-C in this study and other studies in HK, TW, and the USA may have limited comparability given the varied age range and educational levels. Multicenter studies adopting the same inclusion criteria are required in future work for a more convincing comparison.

### Effects of Demographic Factors on the BNT-C Performance

Demographic variables including age, gender, and education were repeatedly reported to impact the performance of cognition measures. Lower mean BNT scores with lower educational levels have been frequently found in the published works of literature (26–30). This study also demonstrated that education correlated significantly with BNT-C scores. Our findings were consistent with other research. Cheung et al. (20) performed the BNT-C in 77 normal adults in Hong Kong and found a positive association between education and naming scores (*r* = 0.342, *P* < 0.01). Chen et al. (5) applied the same BNT-C to 264 native Chinese people with normal cognition aged >60 years in Taiwan. They also found years of education were positively correlated with BNT-C score (*r* = 0.376, *P* < 0.01). Our research further showed that high-educated participants made fewer errors than low-educated subjects on dart, harp, pyramid, tripod, and protractor. The results were supported by the study exploring the influence of schooling on the performance of aphasia examination, which indicated that confrontation naming demands a greater degree of semantic knowledge, which is proved with increasing years of formal education (31). This may be because these objects are not common in daily life but are acquired gradually in study and reading.

The influence of gender and age on BNT performance remains controversial. Using the original 60-item BNT in normal elders in Middle Tennessee, Welch (30) found that age was significantly involved in confrontational naming ability. Moreover, there was also a gender bias that men scored significantly higher on 17 items than women. However, no gender effect was found for the Turkish version of the BNT (32) and the adapted BNT version for the Portuguese speakers (33). The Korean version of the BNT scores was slightly affected by age but rarely influenced by gender (10). For BNT-C, Cheung, who developed the BNT-C, found that gender and age have no effects on naming performance in Hongkong Chinese population (20). Chen et al. (5) did not identify the effect of either gender and age on BNT-C scores in the Taiwan residents. Consistent with previous research, we found no correlations between age or gender and BNT-C performance in this study. It likely indicated that gender and age affect the performance less on this 30-item BNT-C version.

This study has several limitations. First, our study mainly recruited the elders aged ≥55 years, so the data may not be generalized to the adults of all ages. Second, the major subjects were educated and only few were illiterate. This might underestimate the education effects on BNT-C performance. However, with the popularization of compulsory education, there were fewer illiterates now, and there will hardly be illiterates in the future. The results of this study will be suitable for future use. Third, the number of participants in each age-education cell was relatively small. Further research should expand the sample size. Fourth, the participants were recruited from communities in the urban areas of Beijing. In the future, urban and rural residents should be selected nationwide to make the results more representative. Another one that should be considered was that the American, Hong Kong, and Taiwan studies used for comparison were conducted 10 years or more before. The results may deviate from the real differences between the current population. However, the Chinese people rated less familiar and score on many items than the American decades later, indicating that cultural differences between China and America and their impact on BNT-C persist till now.

## Conclusions

To our knowledge, it was the first time to establish norm scores of BNT-C considering influencing factors in the elderly population of Chinese mainland. These scores presented here take into account subjects' education level and are therefore likely to help clinicians make diagnostic decisions more accurately. Further research should expand the sample size and explore the sensitivity and specificity of the BNT-C for the linguistic disorders in the native Chinese-speaking population. We also found a notable difference in participants' familiarity and naming accuracy for each BNT-C item between the Americans and native Chinese speakers, which was highly consistent with the cultural differences. The possibility of replacing items that are not suitable for the Chinese individuals with more culturally representative objects should be considered in future work.

## Data Availability Statement

The raw data supporting the conclusions of this article will be made available by the authors, without undue reservation.

## Ethics Statement

The studies involving human participants were reviewed and approved by Ethics Committee of Xuanwu Hospital, Capital Medical University. The patients/participants provided their written informed consent to participate in this study.

## Author Contributions

AZ: conception, design, and revision of the article. YL, YQ, FW, and CW: data collection. YL: analysis and drafting of the article. RW, HJ, BX, and JY: data collection. JJ: revision of the article and approved the final version. All authors contributed to the article and approved the submitted version.

## Funding

This study was supported by the projects of the National Natural Science Foundation of China (No. 30900394).

## Conflict of Interest

The authors declare that the research was conducted in the absence of any commercial or financial relationships that could be construed as a potential conflict of interest.

## Publisher's Note

All claims expressed in this article are solely those of the authors and do not necessarily represent those of their affiliated organizations, or those of the publisher, the editors and the reviewers. Any product that may be evaluated in this article, or claim that may be made by its manufacturer, is not guaranteed or endorsed by the publisher.
